# First record of theridiosomatid genus *Sennin* Suzuki, Hiramatsu & Tatsuta, 2022 from Anhui Province, China, with the description of a new species (Araneae, Theridiosomatidiae)

**DOI:** 10.3897/BDJ.11.e107528

**Published:** 2023-10-24

**Authors:** Yanbin Yao, Mingkang Liu, Rui Zhao, Zijie Deng, Keke Liu

**Affiliations:** 1 Jinshan College of Fujian Agriculture And Forestry University, Fuzhou, China Jinshan College of Fujian Agriculture And Forestry University Fuzhou China; 2 College of Life Science, Jinggangshan University, Ji'an, China College of Life Science, Jinggangshan University Ji'an China; 3 College of Animal Science, Fujian Agriculture And Forestry University, Fuzhou, China College of Animal Science, Fujian Agriculture And Forestry University Fuzhou China; 4 College of Plant Science & Technology, Huazhong Agriculture University, Wuhan, China College of Plant Science & Technology, Huazhong Agriculture University Wuhan China

**Keywords:** Cave spider, small body size, taxonomy

## Abstract

**Background:**

Only two *Sennin* species are known from the world, *Sennincoddingtoni* (Zhu, Zhang & Chen, 2001) from China and *Sennintanikawai* Suzuki, Hiramatsu & Tatsuta, 2022 from Ryukyu Islands. No other *Sennin* species have been recorded from other locations.

**New information:**

A new species, *Senninshuanglong* **sp. n.** is described from Anhui Province, China. Morphological illustrations, SEMs, living photos, habitat and distribution map are given.

## Introduction

The spider family Theridiosomatidiae Simon, 1881 includes 137 species from 20 genera worldwide (World Spider Catalog 2023). At present, 30 species belonging to 12 genera are known from China (World Spider Catalog 2023). Most of these species (21 species) are distributed in southwest China, but there are few records (2 species) in eastern China (World Spider Catalog 2023). They are a widely distributed family of very small body size (usually ≤ 3 mm) and usually found in damp, dark habitats, such as the litter layer of forest or in caves (Zhao and Li 2012). Although many species have been reported in the past 15 years (World Spider Catalog 2023), there are still many poorly-known theridiosomatid and other species (Liu et al. 2022) from southern China with unusual morphological characteristics.

The genus *Sennin* was established by Suzuki et al. (2022), based on the type species, *Sennintanikawai* Suzuki, Hiramatsu & Tatsuta, 2022 recorded from Ryukyu Islands. One *Karstia* species, recorded from Guizhou Province, China, was transferred to this genus by Suzuki et al. (2022). Now, the genus only consists of these two species mainly recorded from Asia.

In a recent exploration of the limestone caves in Anhui Province, China, one undescribed species of this poorly-known genus was found. The aim of the present paper is to provide a detailed description of this new species whichalso represents the first record of this family from Anhui Province.

## Materials and methods

Specimens were examined using a SZ6100 stereomicroscope. Both male and female copulatory organs were dissected and examined in 80% ethanol using an Olympus CX43 compound microscope with a KUY NICE CCD camera. Epigynes were cleared with pancreatin solution (Álvarez-Padilla and Hormiga 2007). Specimens, including dissected male palps and epigynes, were preserved in 75% ethanol after examination. Types are deposited in the Animal Specimen Museum, College of Life Science, Jinggangshan University (ASM-JGSU).

The measurements were taken using a stereomicroscope (AxioVision SE64 Rel. 4.8.3) and are given in millimetres. The body lengths of all specimens exclude the chelicerae and spinnerets. Terminology of the male and female genitalia follows Suzuki et al. (2022).

The abbreviations used in the figures and text are: ALE − anterior lateral eye, AME − anterior median eye, CA − cymbial apophysis, CB − copulatory bursae, C − conductor, CD − copulatory ducts, CL − cymbial lamella, EA − embolic apophysis, ED − embolic division, E − embolus, ES − epigynal scape, FD − fertilisation ducts, MA − median apophysis, PC − paracymbium, PLE − posterior lateral eye, PME − posterior median eye, S − spermatheca.

## Taxon treatments

### Sennin shuanglong

Yao & Liu, 2023 sp. nov.

F3CB2093-3D75-56F6-81FB-D58C0C44784E

7F73E688-F2EF-40E6-B11D-222F4B56A6B5

#### Materials

- **Type status:** Holotype. **Occurrence:** recordedBy: Liu Ke-Ke; individualCount: 1; sex: male; lifeStage: adult; occurrenceID: FD0ADBD2-F466-5410-B79D-412D4C28B029; **Taxon:** scientificName: *Senninshuanglong* Yao & Liu, sp. n.; **Location:** country: China; stateProvince: Anhui; locality: Tongling City, Yi’an District, Tianmen Town, Shuanglong Cave; verbatimCoordinates: 30°51'18.65"N, 117°50'52.71"E; georeferenceProtocol: Baidu Map; **Event:** samplingProtocol: handing; eventDate: 28/06/2022
- **Type status:** Paratype. **Occurrence:** recordedBy: Liu Ke-Ke; individualCount: 4; sex: male; lifeStage: adult; occurrenceID: 8DBABF2F-D78A-509E-93C0-91F9985E8D5E; **Taxon:** scientificName: *Senninshuanglong* Yao & Liu, sp. n.; **Location:** country: China; stateProvince: Anhui; locality: Tongling City, Yi’an District, Tianmen Town, Shuanglong Cave; verbatimCoordinates: 30°51'18.65"N, 117°50'52.71"E; georeferenceProtocol: Baidu Map; **Event:** samplingProtocol: handing; eventDate: 21/12/2022-03/02/2023
- **Type status:** Paratype. **Occurrence:** recordedBy: Liu Ke-Ke; individualCount: 14; sex: female; lifeStage: adult; occurrenceID: B5DD73C1-24C4-5FF5-B1D9-0B215821DA96; **Taxon:** scientificName: *Senninshuanglong* Yao & Liu, sp. n.; **Location:** country: China; stateProvince: Anhui; locality: Tongling City, Yi’an District, Tianmen Town, Shuanglong Cave; verbatimCoordinates: 30°51'18.65"N, 117°50'52.71"E; georeferenceProtocol: Baidu Map; **Event:** samplingProtocol: handing; eventDate: 21/12/2022-03/02/2023

#### Description

**Figure 1.**
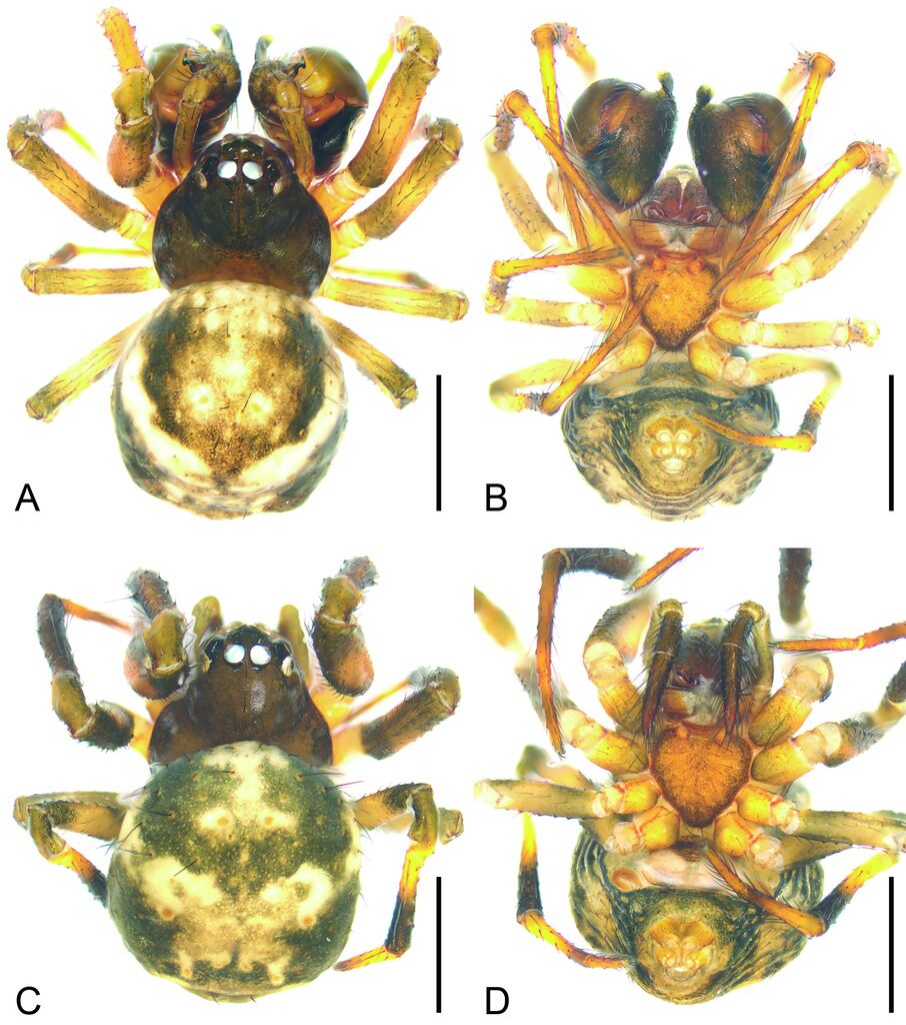
*Senninshuanglong* sp. n., habitus of male holotype and female paratype. **A, C** habitus, dorsal view; **B, D** same, ventral view. Scale bars: 1 mm.

**Figure 2.**
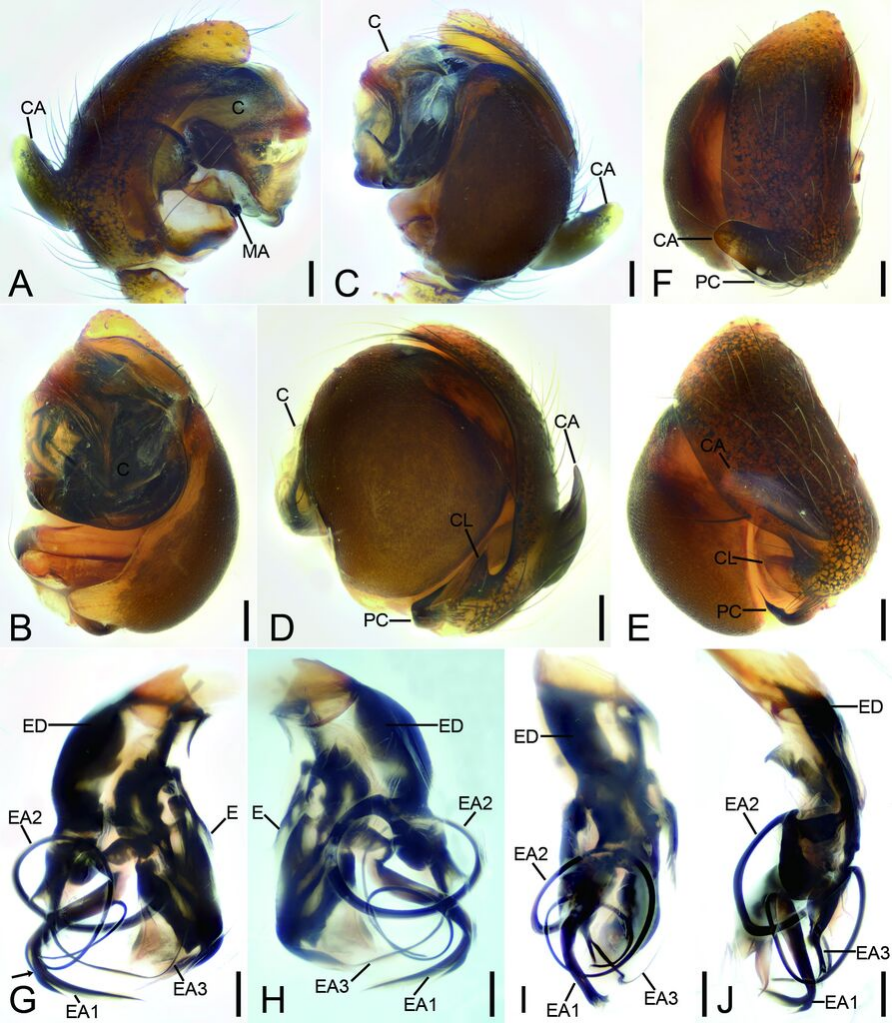
*Senninshuanglong* sp. n., male palp. **A** palp of holotype, prolateral view; **B** same, ventral view; **C** same, anterior view; **D** same, retrolateral view; **E** same, dorsal view; **F** same, superjacent view; **G** palp of paratype, ventral view, black arrow shows the bifurcate tip of EA1; **H** same, anterior-dorsal view; **I** same, prolateral view; **J** same, posterior view, black arrow shows the bifurcate tip of EA1. Abbreviations: **C**−conductor, **CA**−cymbial apophysis, **CL**−cymbial lamella, **E**−embolus, **EA**−embolic apophysis, **ED**−embolic division, **MA**−median apophysis, **PC**−paracymbium. Scale bars: 0.1 mm.

Male (Fig. 1A, B, Fig. 2, Fig. 4A−K and Fig. 5D). Measurements. Body 2.31 long. Carapace oval, with sparse setae on dorsal surface, 0.95 long, 1.07 wide. Eyes: with black annulations; measurements: AME 0.12, ALE 0.11, PME 0.12, PLE 0.10, AME−AME 0.05, AME−ALE 0.04, PME−PME 0.02, PME−PLE 0.08, AME−PME 0.11, AME−PLE 0.18, ALE−ALE 0.31, PLE−PLE 0.38, ALE−PLE, 0.02. Chelicerae stout, with five promarginal teeth, a single retromarginal tooth and 45 small denticles in between the teeth. Endites wider than long. Labium triangular, slightly shorter than endites. Sternum sub-triangular, as long as wide, posterior end blunt. Leg measurements: leg I: 1.23+0.47+1.00+0.64+0.64 = 3.98; leg II: 0.97+0.34+0.77+0.63+0.51 = 3.22; leg III: 0.73+0.27+0.56+0.42+0.36 = 2.34; leg IV: 0.92+0.30+0.58+0.43+0.40 = 2.63. Abdomen ovoid, covered with long and thin setae, 1.21 long, 1.35 wide.

Colouration (Fig. 1A and B). Carapace black brown. Chelicerae yellow brown. Endites yellow. Labium, anterior part pale, posterior yellow. Sternum yellow, mottled, with abundant black spots. Legs with black brown annulations on femora, tibiae and metatarsi. Abdomen mottled, with a pale ring-shaped band; venter black brown.

Palp (Fig. 2 and Fig. 4A−K). Cymbium: cymbial apophysis finger-like, slightly shorter than cymbial width in dorsal view; cymbial lamella triangular, with blunt and cone-shaped tip in prolateral view, like a barb in retrolateral view; paracymbium hook-shaped, with needle-like tip in retrolateral view. Median apophysis small hook-shaped, with a broad base, apex curved forward. Conductor translucent, covering complex embolic system. Embolus: embolic division complex, with multiple slender protrusions; embolus embranous, nearly as long as EA3, covered with a membrane, including three apophyses; EA 1 thickest, C-shaped, with broad membranous apex, through EA 2 and EA 3; EA 2 very long, filiform, spiralling around EA 1, with sharp apex; EA 3 flagelliform, lamellar, with two basal apophyses, one clavate, the other tooth-like.

**Figure 3.**
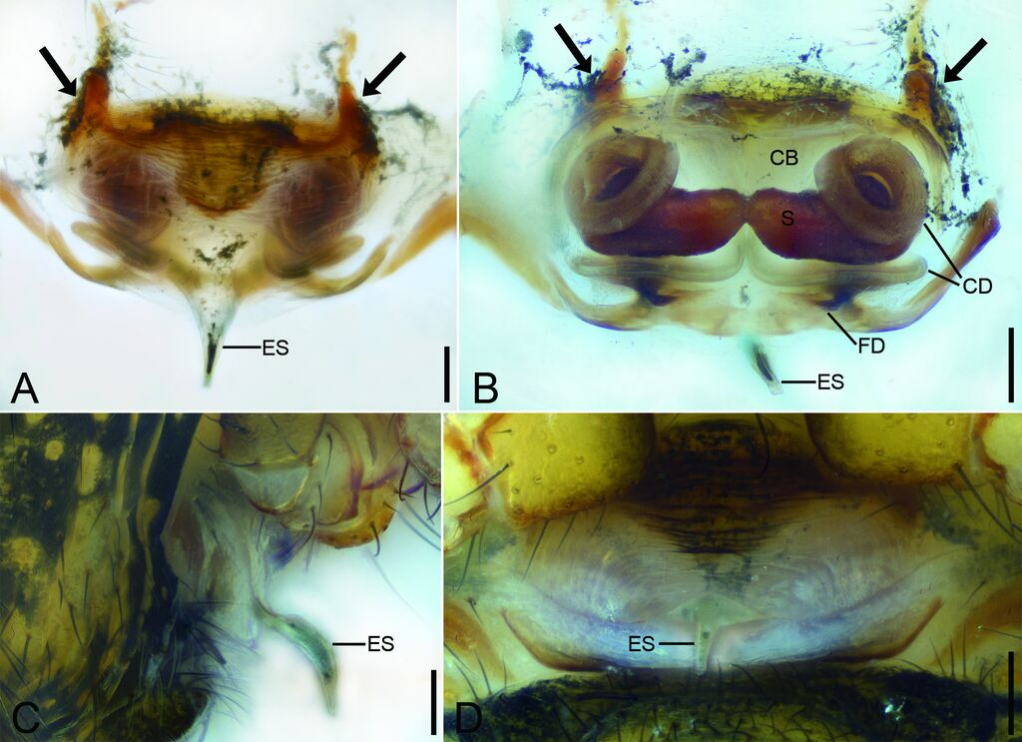
*Senninshuanglong* sp. n., epigyne of female paratype. **A** epigyne, ventral view, black arrow shows a pair of sclerotised extensions on the anterior margin of epigynal plate; **B** vulva, dorsal view; **C** epigyne, lateral view; **D** epigyne, ventral view. Abbreviations: **CB**−copulatory bursae, **CD**−copulatory ducts, **ES**−epigynal scape, **FD**−fertilisation ducts, **S**−spermatheca. Scale bars: 0.1 mm.

**Figure 4.**
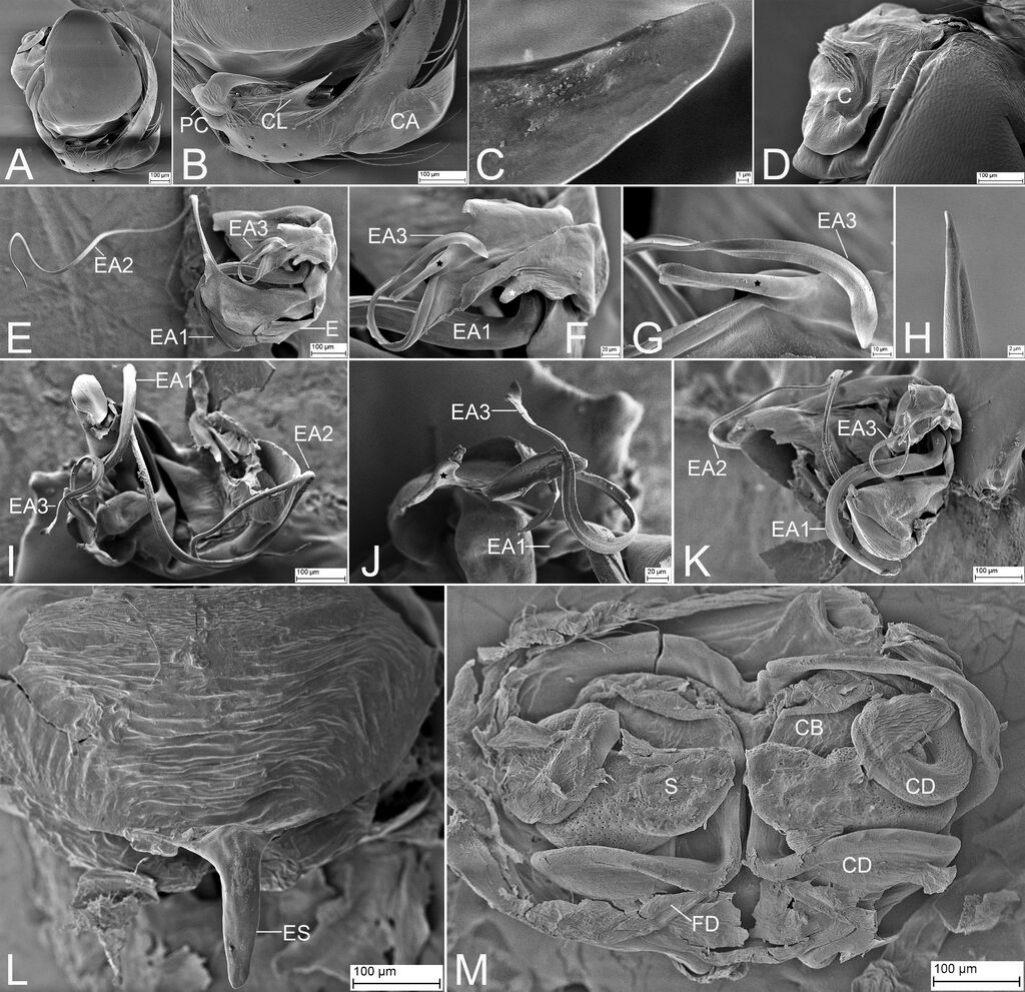
*Senninshuanglong* **sp. n.**, male palp and female epigyne, paratype. **A** palp, retrolateral view; **B** same, detail of CL, PC and CA, retrolateral view; **C** same, detail of the apex of CL, retrolateral view; **D** same, detail of conductor, retrolateral view; **E** embolic division, ventral view; **F** same, detail of EA3, black and white stars show the basal apophyses of EA3, ventral view; **G** same, detail of apex of EA3, black star shows the basal apophysis of EA3, ventral view; **H** same, detail of the apex of EA2, ventral view; **I** same, posterior-dorsal view; **J** same, detail of EA1 and EA3, black star shows the basal apophysis of EA3, posterior-dorsal view; **K** same, detail of EA1, EA2 and EA3, posterior-dorsal view; **L** epigyne, ventral view; **M** same, dorsal view. Abbreviations: **C**−conductor, **CA**−cymbial apophysis, **CB**−copulatory bursae, **CD**−copulatory ducts, **CL**−cymbial lamella, **E**−embolus, **EA**−embolic apophysis, **ES**−epigynal scape, **FD**−fertilisation ducts, **MA**−median apophysis, **PC**−paracymbium, **S**−spermatheca.

**Figure 5.**
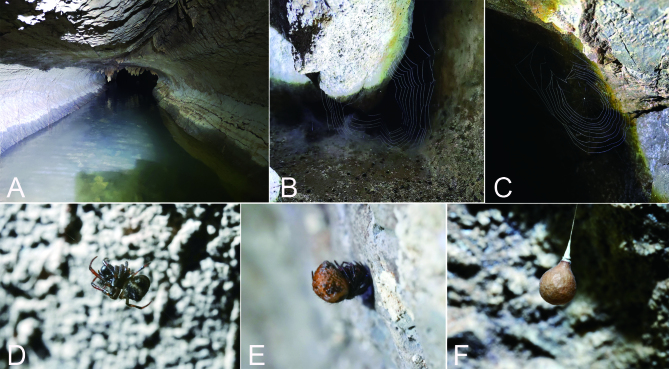
*Senninshuanglong* sp. n. **A** habitat, aspect of Shuanglong limestone cave; **B, C** webs; **D** male; **E** female; **F** egg sac.

Female (Fig. 1C, D, Fig. 3, Fig. 4L, M and Fig. 5E). As in male, except as noted. Measurements. Body 2.46 long. Carapace 1.26 long, 1.14 wide. Abdomen 1.77 long, 1.66 wide. Eye size and measurements: AME 0.14, ALE 0.13, PME 0.13, PLE 0.11, AME−AME 0.04, AME−ALE 0.05, PME−PME 0.04, PME−PLE 0.09, AME−PME 0.12, AME−PLE 0.19, ALE−ALE 0.34, PLE−PLE 0.42, ALE−PLE, 0.04. Leg measurements: leg I: 1.25+0.45+0.87+0.72+0.50 = 3.79; leg II: 1.05+0.35+0.73+0.60+0.47 = 3.20; leg III: 0.74+0.33+0.47+0.47+0.34 = 2.35; leg IV: 0.76+0.41+0.65+0.51+0.38 = 2.71.

Colouration (Fig. 1C and D). Lighter than male. Abdomen lacking ring-like white band, with two pairs of white patches in anterior half part.

Epigyne (Fig. 3, Fig. 4L and M). Epigynal plate wider than long, posteriorly with a protruding, long and banana-shaped epigynal scape, convex ventrally. Copulatory opening very small. Copulatory bursae developed, membranous, touching. Copulatory ducts originating from copulatory bursae, extending along the mesial line of the vulva, running posterior-dorsally under spermathecae, bent at an acute angle towards anteromedially, curving backwards at lateral side of spermathecae, spiralling a circle. Spermathecae located medially, moderate tapering in touching part. Fertilisation ducts short, running under copulatory ducts, medially directed.

#### Diagnosis

Males of this species is similar to that of *Sennintanikawai* Suzuki, Hiramatsu & Tatsuta, 2022 in having the finger-like cymbial apophysis in dorsal view and the triangular cymbial lamella in retrolateral view (Suzuki et al. 2022: 86, figs. 7A and C), but can be distinguished from it by the median apophysis with a thick, strong curved apex (vs. thin, slightly curved in *S.tanikawai*) and the paracymbium with a sharp needle-like apex (vs. spine-like in *S.tanikawai*). It also resembles *S.coddingtoni* (Zhu, Zhang & Chen, 2001) in the triangular cymbial lamella, but can be easily separated from it by the large cymbial apophysis (vs. small) (Chen 2010: 8, fig. 27) (Fig. 2D, E, Fig. 4A and B). Females resemble those of *S.coddingtoni* (Chen 2010: 7, figs. 19 and 20) and *S.tanikawai* (Suzuki et al. 2022: 87, fig. 8) in having the copulatory duct with a coil laterally located, but can be distinguished from it by the very long epigynal scape as same as epigynal length (vs. relative long epigynal scape shorter than epigynal length in *S.coddingtoni* and *S.tanikawai*) and the transversal spermathecae with a tapering tip in touching area (vs. the transversal spermathecae without tapering tip; the sloping spermathecae in *S.tanikawai*) (Fig. 3, Fig. 4L and M).

#### Etymology

The specific name is a noun in apposition and refers to the type locality.

#### Distribution

**Figure 6.**
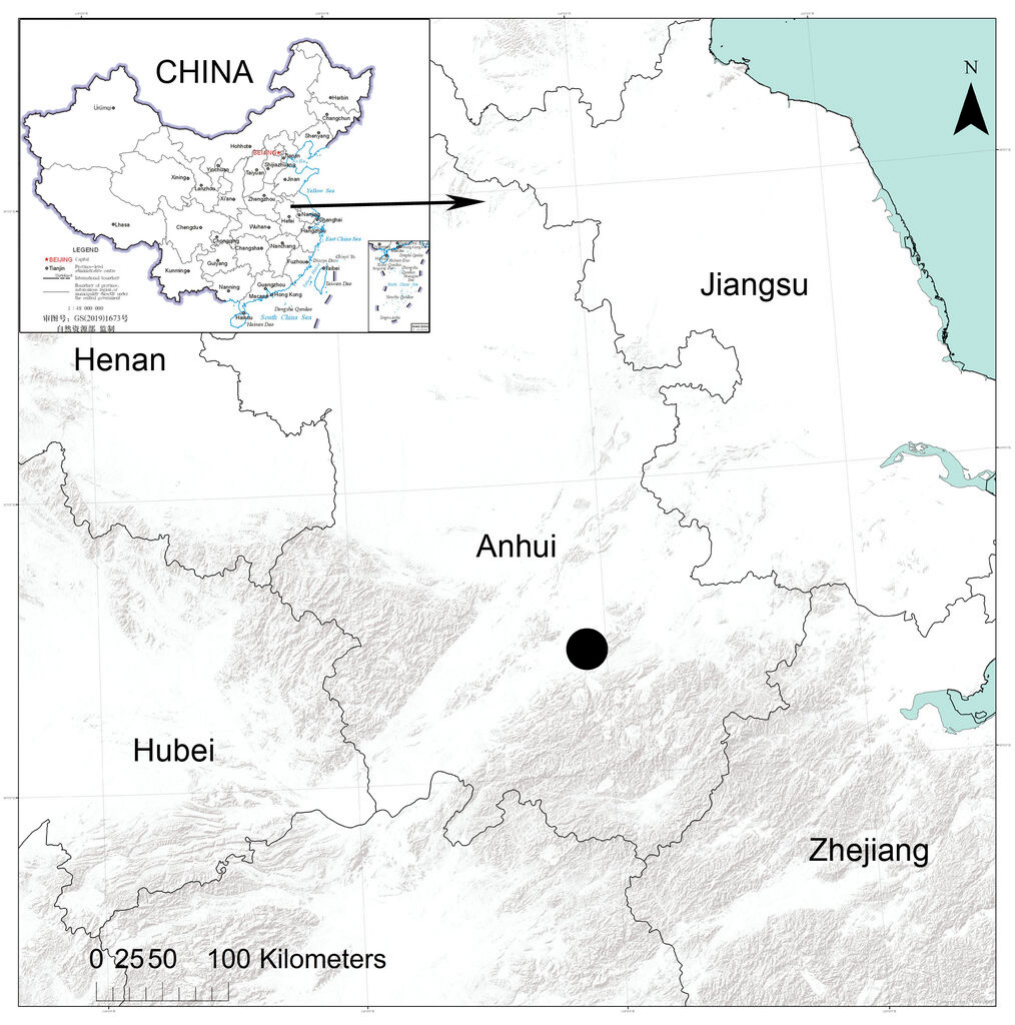
Records of *Senninshuanglong* sp. n. from Anhui Province, China.

Known only from the type locality in Anhui Province, China (Fig. 6).

#### Ecology

The new species only inhabits deep within limestone caves (Fig. 5A). These spiders build vertical circular webs with the junction of the top and sides of the cave (Fig. 5B and C). Egg sacs (Fig. 5F) spherical, are suspended with a long vertical line on the roof of the cave near female webs.

## Supplementary Material

XML Treatment for Sennin shuanglong
